# Rescue of Holoprosencephaly in Fetal Alcohol-Exposed *Cdon* Mutant Mice by Reduced Gene Dosage of *Ptch1*

**DOI:** 10.1371/journal.pone.0079269

**Published:** 2013-11-11

**Authors:** Mingi Hong, Robert S. Krauss

**Affiliations:** 1 Department of Developmental and Regenerative Biology, Icahn School of Medicine at Mount Sinai, New York, New York, United States of America; Columbia University, United States of America

## Abstract

Holoprosencephaly (HPE) is a commonly occurring developmental defect in which midline patterning of the forebrain and midface is disrupted. Sonic hedgehog (SHH) signaling is required during multiple stages of rostroventral midline development, and heterozygous mutations in SHH pathway components are associated with HPE. However, clinical presentation of HPE is highly variable, and carriers of heterozygous mutations often lack apparent defects. It is therefore thought that such mutations must interact with more common modifiers, genetic and/or environmental. We have modeled this scenario in mice. *Cdon* mutant mice have a largely subthreshold defect in SHH signaling, rendering them sensitive to a wide spectrum of HPE phenotypes by additional hits that are themselves insufficient to produce HPE, including transient in utero exposure to ethanol. These variable HPE phenotypes may arise in embryos that fail to reach a threshold level of SHH signaling at a specific developmental stage. To provide evidence for this possibility, here we tested the effect of removing one copy of the negative regulator *Ptch1* from *Cdon^−/−^* embryos and compared their response to ethanol with that of *Cdon^−/−^;Ptch1^+/+^* embryos. *Ptch1* heterozygosity decreased the penetrance of HPE in this system by >75%. The major effect of reduced *Ptch1* gene dosage was on penetrance, as those *Cdon^−/−^;Ptch1^+/−^* embryos that displayed HPE did not show major differences in phenotype from *Cdon^−/−^;Ptch1^+/+^* embryos with ethanol-induced HPE. Our findings are consistent with the notion that even in an etiologically complex model of HPE, the level of SHH pathway activity is rate-limiting. Furthermore, the clinical outcome of an individual carrying a SHH pathway mutation will likely reflect the sum effect of both deleterious and protective modifier alleles and their interaction with non-genetic risk factors like fetal alcohol exposure.

## Introduction

Holoprosencephaly (HPE), a common developmental defect of the forebrain and midface, results from disruption in patterning of the rostroventral midline [1]. HPE occurs with high frequency, ∼1∶250 human conceptions, the great majority of which are lost during pregnancy [2], [3]. Both genetic and environmental factors are implicated in the etiology of HPE [4]–[6]. Heterozygous mutations in components of the Sonic hedgehog (SHH) signaling pathway are found in both inherited and sporadic forms of HPE [5]. These include the SHH ligand itself and the receptors, PTCH1, CDON, and GAS1 [5], [7], [8]. Among the non-genetic factors suggested to elevate the risk of HPE are preexisting maternal diabetes and fetal alcohol exposure [9], [10].

Experiments with mice and other vertebrate model organisms indicate that development of the rostroventral midline occurs by a progressive patterning mechanism whereby SHH produced by one midline structure induces *Shh* expression in a successive midline structure. SHH produced by the prechordal mesendoderm (PCM) is a key early step in development of the midline of the forebrain and midface [1], [11]–[13]. SHH produced by the PCM induces expression of pathway target genes (including *Shh* itself) in the rostral diencephalon ventral midline of the developing forebrain, which in turn leads to *Shh* expression in the ventral telencephalon [13]–[18]. As development proceeds, SHH produced by the forebrain induces expression of *Shh* in the ectoderm of the frontonasal and maxillary processes of the developing face, helping to pattern the craniofacial midline [17], [19]–[21].

Clinical presentation of HPE is extremely variable, and the range of defects falls within a continuum known as the HPE spectrum [4], [22]. The most severe form, alobar HPE, is characterized by complete failure to partition the forebrain into left and right hemispheres; this is usually associated with the most severe midfacial phenotypes, including cyclopia. The spectrum encompasses progressively less severe forebrain defects (semilobar and lobar HPE) and also mild facial midline abnormalities (so-called HPE microforms) that can occur in the absence of brain malformations.

A full spectrum of HPE phenotypes is seen both in sporadic and familial cases [23]. Furthermore, many mutation carriers in pedigrees are without apparent clinical manifestation, and mutations in many sporadic HPE patients are inherited from an apparently unaffected parent [23], [24]. Statistical analysis of these observations and gene mutation frequencies have led to an “autosomal dominant with modifier” model, in which the phenotypic outcome associated with a heterozygous mutation is influenced by more common genetic variants and/or environmental exposures [25]. It is important, therefore, to identify interactions between bona fide mutations and additional factors that grade penetrance and expressivity, resulting in the wide range of defects encompassed by the HPE spectrum. Animal models are well placed to do so. We have established *Cdon* mutant mice as such a model. Mice lacking CDON on a 129S6 genetic background have a largely subthreshold defect in SHH signaling that renders them sensitive to induction of HPE by second hits that are, themselves, insufficient to produce HPE (e.g., removal of one copy of *Shh*; gene dosage-sensitive removal of the *Cdon* paralog, *Boc*) [26]–[28].

We recently reported use of these mice to develop a model of gene-environment interaction in HPE. In utero ethanol exposure at E7.0 had little or no effect on wild type 129S6 embryos, but produced a wide range of HPE phenotypes with >75% penetrance in *Cdon^−/−^* embryos [29]. The spectrum of HPE phenotypes produced in ethanol-treated *Cdon^−/−^* mice ranged from alobar HPE with an undivided eye field, to lobar HPE with single nostril, to microform HPE and defects in palatogenesis [29]. A model consistent with production of phenotypes at both the severe and mild ends of the spectrum in ethanol-treated 129S6.*Cdon^−/−^* mice is as follows: ethanol initiates defects in midline patterning specifically in genetically sensitized embryos; the variable severity of the HPE phenotype may arise as a consequence of individual embryos failing to reach a threshold level of SHH signaling at specific developmental stages, the stage of the deficit occurring stochastically and dictating phenotypic severity [29]. This hypothesis argues that pathway signaling strength is an important determinant of phenotypic outcome. PTCH1, the primary SHH receptor, functions as a negative regulator of pathway signaling [30]. To test the hypothesis, we removed one copy of *Ptch1* from 129S6.*Cdon^−/−^* embryos and assessed ethanol-induced production of HPE. As predicted by the model, *Ptch1* heterozygosity effectively rescued such embryos.

## Results

### Rescue of HPE in fetal alcohol-exposed *Cdon* mutant mice by reduced *Ptch1* gene dosage

Loss of *Cdon* or in utero ethanol exposure in 129S6 mice gave little or no phenotype individually, but together produced defects in midline patterning, inhibition of SHH signaling in the developing forebrain, and a broad spectrum of HPE phenotypes [29]. When analyzed at E10.0, ∼13.5% of ethanol-treated 129S6.*Cdon^−/−^* embryos displayed severe phenotypes that included alobar HPE and failure to divide the eye field. These embryos died in utero and were resorbed by E11.0. When analyzed at E14.0, ∼70% of 129S6.*Cdon^−/−^* embryos displayed phenotypes ranging from lobar through microform HPE, and variously included single nostril, defective palatogenesis, diminished nasal septal cartilage, and rudimentary vomeronasal organs. In asking whether removal of one copy of *Ptch1* could rescue HPE in these animals, we analyzed E14.0 embryos to take advantage of the high penetrance of the phenotypes observed at this stage.

Mouse strain-dependent modifier genes regulate HPE phenotypes associated with *Cdon* mutation [27], [28]. Because our ethanol studies have been performed on a congenic 129S6 background, we first generated a congenic 129S6.*Ptch1^+/−^* line by back-crossing. These mice were bred with 129S6.*Cdon^+/−^* mice to generate double heterozygotes. Various offspring of intercrosses of these animals were further crossed, and pregnant females received IP injections of ethanol or saline control at E7.0 and four hours later ([29] and see Materials and Methods). Embryos of four genotypes were studied: *Cdon^+/+^;Ptch1^+/+^*, *Cdon^+/+^;Ptch1^+/−^*, *Cdon^−/−^;Ptch1^+/+^*, and *Cdon^−/−^;Ptch1^+/−^*.

**Figure 1:**
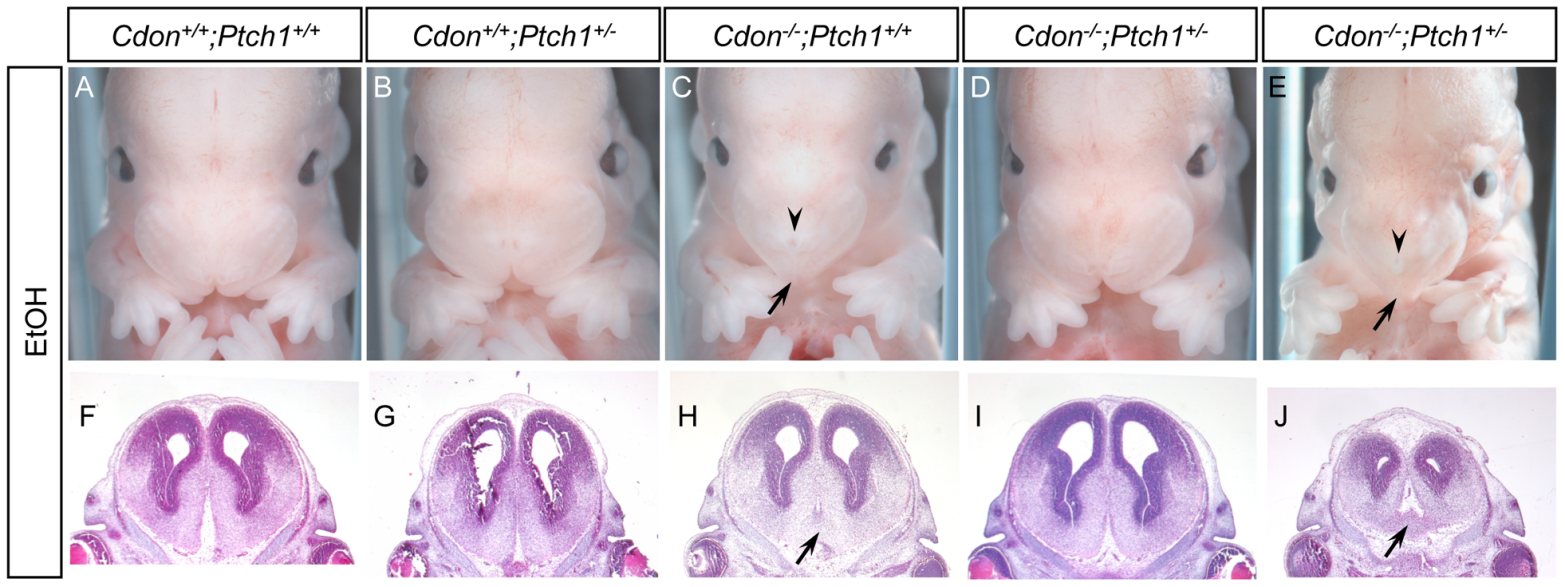
Effect of reduced *Ptch1* gene dosage on ethanol-induced HPE in *Cdon^−/−^* embryos: external features and lobar HPE. (A–E) Frontal views of E14.0 embryos. About 65% of ethanol (EtOH)-treated *Cdon^−/−^;Ptch1^+/+^* embryos displayed strong facial features of HPE, including a single nostril (arrowhead in C) and smooth, pointed philtrum (arrow in C). In contrast, only ∼15% of EtOH-treated *Cdon^−/−^;Ptch1^+/−^* embryos had external features of HPE. Two ethanol-treated *Cdon^−/−^;Ptch1^+/−^* embryos are shown, one that did not have external HPE features (D) and one that did (E; note the single nostril (arrowhead) and smooth, pointed philtrum (arrow)). (F-J) H&E-stained coronal sections of E14.0 embryos. Note that the ethanol-treated *Cdon^−/−^;Ptch1^+/+^* embryo (H) displays continuity across the ventral midline of the forebrain (arrow), indicative of lobar HPE. Two ethanol-treated *Cdon^−/−^;Ptch1^+/−^* embryos are shown, one that did not have external HPE features (I) and one that did (J). Note that only the embryo that had external HPE (J) also had continuity across the ventral midline of the forebrain (arrow). See Figure S1 for saline-treated control embryos. See Table 1 for quantification.

Whole embryos were initially analyzed at E14.0 for external HPE features, including single nostril, deficient philtrum, and hypotelorism. *Cdon^+/+^;Ptch1^+/+^* and *Cdon^+/+^;Ptch1^+/−^* embryos with or without ethanol did not have HPE, nor did saline-treated *Cdon^−/−^;Ptch1^+/+^* and *Cdon^−/−^;Ptch1^+/−^* embryos (Figures 1 and S1, Table 1). Approximately 65% of ethanol-treated *Cdon^−/−^;Ptch1^+/+^* embryos displayed external HPE phenotypes, similar to our previous results. In contrast, only ∼15% of ethanol-treated *Cdon^−/−^;Ptch1^+/−^* embryos displayed HPE, significantly lower than for *Cdon^−/−^;Ptch1^+/+^* embryos (Figure 1, Table 1). Therefore, reduced gene dosage of *Ptch1* substantially, though partially, rescued HPE associated with the synergistic interaction of *Cdon* mutation and fetal alcohol exposure.

**Table 1:** Frequency of HPE defects in ethanol-treated mice.

Defect	Treatment	Genotype (# affected/total (%))			
		*Cdon^+/+^;Ptch1^+/+^*	*Cdon^+/+^;Ptch1^+/−^*	*Cdon^−/−^;Ptch1^+/+^*	*Cdon^−/−^;Ptch1^+/−^*
External HPE features	Saline	0/9	0/13	0/16	0/15
	Ethanol	0/21	0/16	15/23 (65.2%)	4/26 (15.4%)*
					
Lobar HPE	Saline	0/4	0/4	0/4	0/4
	Ethanol	0/4	0/4	2/4	1/3^1^ 0/3^2^
Defective palatogenesis	Saline	0/4	0/4	0/4	0/4
	Ethanol	0/4	0/4	4/4	2/3^1^ 0/3^2^
Diminished nasal septal cartilage and vomeronasal organ	Saline	0/4	0/4	0/4	0/4
	Ethanol	0/4	0/4	4/4	3/3^1^ 0/3^2^
Narrow midface with additional presumptive mesenchyme	Saline	0/4	0/4	0/4	0/4
	Ethanol	0/4	0/4	4/4	3/3^1^ 0/3^2^

* p<0.0005, when comparing ethanol-treated *Cdon^−/−^;Ptch1^+/−^* embryos with ethanol-treated *Cdon^−/−^;Ptch1^+/+^* embryos with a two-tailed Fisher's exact test.

1 These *Cdon^−/−^;Ptch1^+/−^* embryos displayed external HPE features.

2 These *Cdon^−/−^;Ptch1^+/−^* embryos lacked external HPE features.

**Figure 2:**
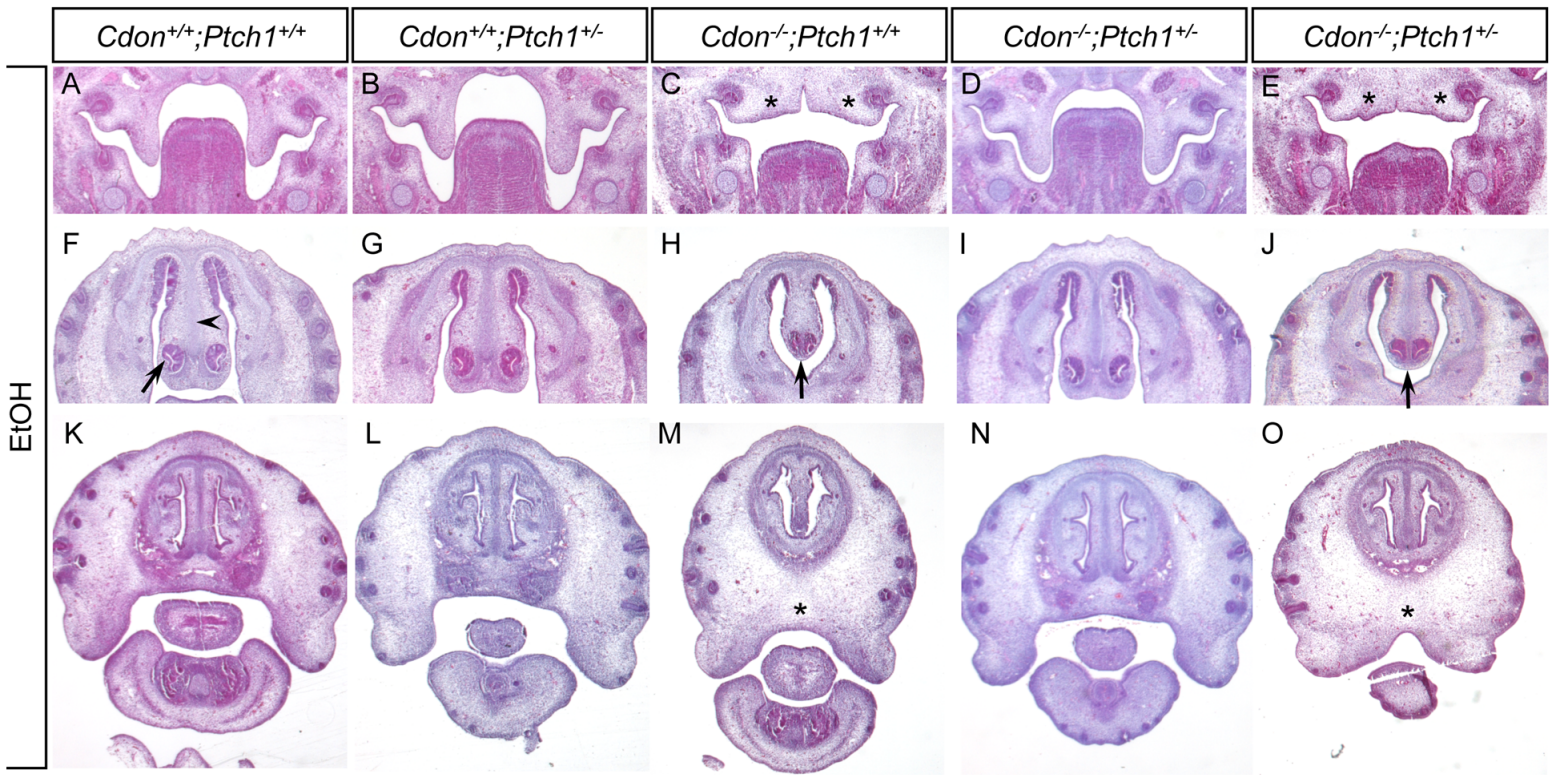
Effect of reduced *Ptch1* gene dosage on ethanol-induced HPE in *Cdon^−/−^* embryos: palatogenesis and midfacial features. (A-O) H&E-stained coronal sections of E14.0 embryos. (A–E) Palatogenesis. Note that the ethanol-treated *Cdon^−/−^;Ptch1^+/+^* embryo (C) displays defective development of the palatal shelves (asterisks). Two ethanol-treated *Cdon^−/−^;Ptch1^+/−^* embryos are shown, one that did not have external HPE features (D) and one that did (E). Note that only the embryo that had external HPE (E) also had defective palatogenesis (asterisks). (F–J) The nasal septal cartilage and vomeronasal organ are denoted in (F) by an arrowhead and arrow, respectively. Note that the ethanol-treated *Cdon^−/−^;Ptch1^+/+^* embryo (H) displays diminished nasal septal cartilage and vomeronasal organ (arrow). Two ethanol-treated *Cdon^−/−^;Ptch1^+/−^* embryos are shown, one that did not have external HPE features (I) and one that did (J). Note that only the embryo that had external HPE (J) also had reduced nasal septal cartilage and vomeronasal organ (arrow). (K–O) Note that the ethanol-treated *Cdon^−/−^;Ptch1^+/+^* embryo (M) displays a narrow midfacial region with additional presumptive mesenchyme (asterisk). Two ethanol-treated *Cdon^−/−^;Ptch1^+/−^* embryos are shown, one that did not have external HPE features (N) and one that did (O). Note that only the embryo that had external HPE (O) also had a narrow midfacial region with additional presumptive mesenchyme (asterisk). See Figure S2 for saline-treated control embryos. See Table 1 for quantification.

Hematoxylin and eosin (H&E)-stained sections of E14.0 embryos were examined to identify defects in forebrain and midline craniofacial structures. We compared four ethanol-treated *Cdon^−/−^;Ptch1^+/+^* embryos that had external HPE features with six ethanol-treated *Cdon^−/−^;Ptch1^+/−^* embryos, 50% of which had external HPE. Saline-treated embryos of these genotypes, as well as saline- and ethanol-treated *Cdon^+/+^;Ptch1^+/+^* and *Cdon^+/+^;Ptch1^+/−^* embryos, all lacked external HPE features and were used as controls (n = 4 for each). As expected [29], these controls showed normal forebrain and craniofacial patterning (Figures 1, 2, S1, and S2, Table 1). Two of the four ethanol-treated *Cdon^−/−^;Ptch1^+/+^* embryos displayed lobar HPE, whereas one of six ethanol-treated *Cdon^−/−^;Ptch1^+/−^* embryos did so (Figure 1F-J, Table 1). All four of the ethanol-treated *Cdon^−/−^;Ptch1^+/+^* embryos had defective palatogenesis, whereas two of six ethanol-treated *Cdon^−/−^;Ptch1^+/−^* embryos did so (Figure 2A-E, Table 1). All four of the ethanol-treated *Cdon^−/−^;Ptch1^+/+^* embryos had diminished nasal septal cartilage and vomeronasal organs; three of the six ethanol-treated *Cdon^−/−^;Ptch1^+/−^* embryos had deficits in these structures (Figure 2F-J, Table 1). Finally, all four ethanol-treated *Cdon^−/−^;Ptch1^+/+^* embryos had narrow midfacial regions that also contained additional presumptive mesenchymal tissue (Figure 2K-O, Table 1), a phenotype seen in *Cdon* mutants of a mixed genetic background [31]. Three of six ethanol-treated *Cdon^−/−^;Ptch1^+/−^* also showed this phenotype (Figure 2K-O, Table 1). A critical point here is that the ethanol-treated *Cdon^−/−^;Ptch1^+/−^* embryos that displayed defects in forebrain and midline craniofacial structures in sections were always among the three that showed external features of HPE; none of the ethanol-treated *Cdon^−/−^;Ptch1^+/−^* embryos that lacked external HPE features showed abnormalities upon sectioning (Table 1).

Taken together, there may be a small diminution in HPE phenotypic expressivity in ethanol-treated *Cdon^−/−^;Ptch1^+/−^* embryos, relative to similarly treated *Cdon^−/−^;Ptch1^+/+^* embryos. However, in most cases, embryos of either genotype that displayed external HPE features showed roughly similar defects in forebrain and midfacial regions. These results suggest that the major effect of removing one copy of *Ptch1* is to decrease the penetrance of HPE in ethanol-treated *Cdon^−/−^* mice, rather than to selectively alter the severity of some HPE phenotypes and not others.

### Effect of *Ptch1* gene dosage on *Shh* and *Nkx2.1* expression in the ventral forebrain of ethanol-treated *Cdon^−/−^* mice

**Figure 3:**
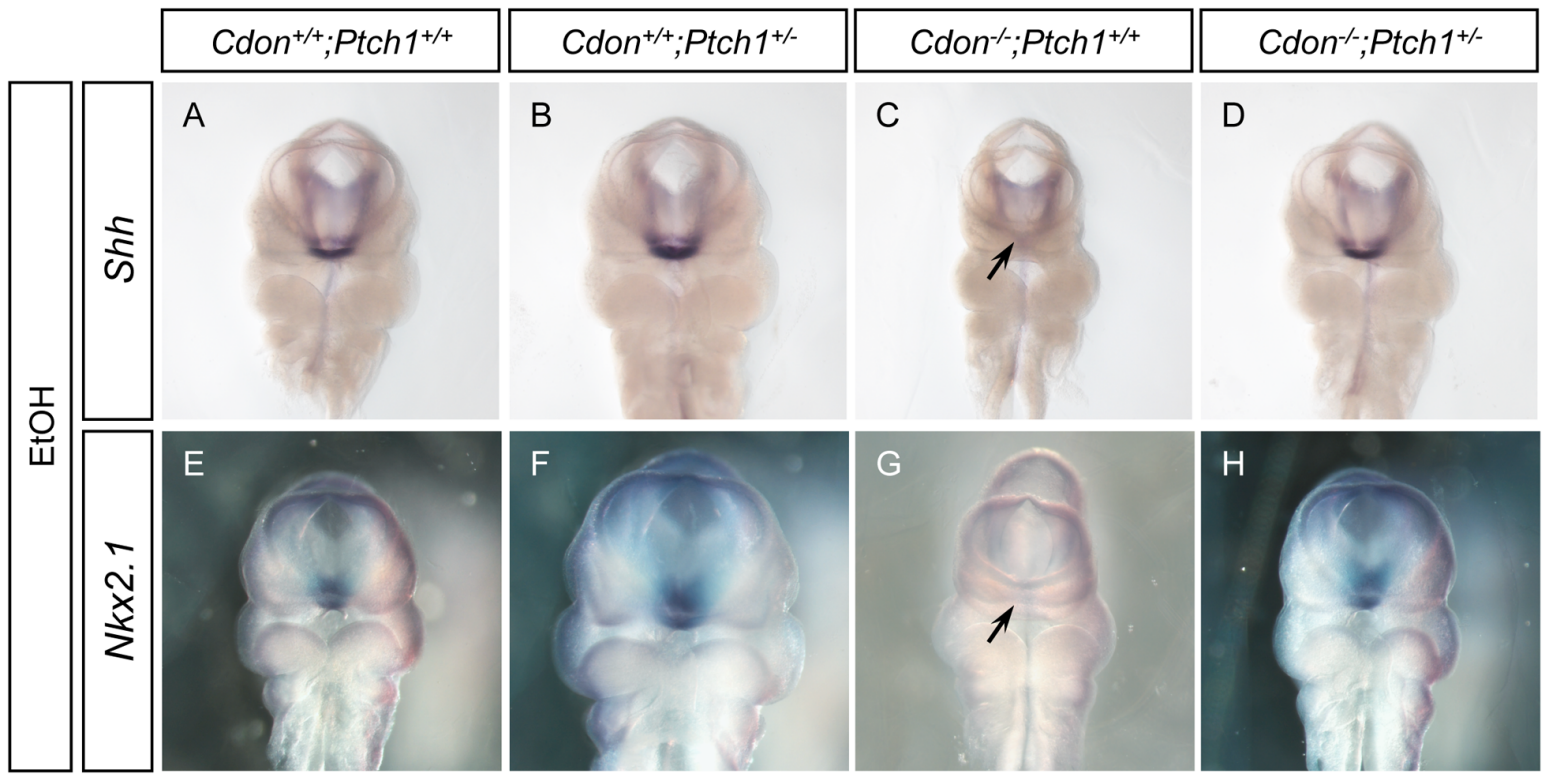
Reduced *Ptch1* gene dosage rescued defective expression of *Shh* and *Nkx2.1* in ethanol-induced HPE in *Cdon^−/−^* embryos. Whole mount in situ hybridization analyses of *Shh* (A–D) and *Nkx2.1* (E–H) expression in E10.25 (31–35 somites) ethanol-treated embryos of the indicated genotype. Three out of eight and five out of nine ethanol-treated *Cdon^−/−^;Ptch1^+/+^* embryos showed defective *Shh* and *Nkx2.1* expression in the forebrain, respectively (arrows in C and G). None of the ethanol-treated *Cdon^−/−^;Ptch1^+/−^* embryos displayed alterations in *Nkx2.1* expression (n = 7), and only one of eight such embryos showed reduced *Shh* expression. See Figure S3 for saline-treated control embryos.

We next tested the expression of *Shh* and a direct SHH pathway target gene, *Nkx2.1*, in the forebrain by whole-mount in situ hybridization at E10.25. We previously found that about half of the ethanol-treated *Cdon^−/−^* embryos at this stage had significantly reduced expression of these genes in the developing ventral telencephalon [29]. In this set, we observed that three of eight ethanol-treated *Cdon^−/−^;Ptch1^+/+^* embryos had an obvious diminution in *Shh* expression and five of nine embryos of this genotype had an obvious reduction in *Nkx2.1* expression (Figure 3). In contrast, one of eight ethanol-treated *Cdon^−/−^;Ptch1^+/−^* embryos showed a noticeable change in the *Shh* expression pattern and no *Cdon^−/−^;Ptch1^+/−^* embryos had alterations in the expression pattern *of Nkx2.1*, as compared to controls (n = 7) (Figures 3 and S3).

## Discussion

HPE is etiologically complex, involving interactions between the timing and strength of developmental signaling pathways, genetic variation, and exposure to environmental agents [4], [22]. The preponderance of the evidence is consistent with an “autosomal dominant plus modifier” model, wherein inherited or de novo heterozygous mutations in one of at least 12 genes interact with more common modifier loci and/or environmental exposures to generate a wide spectrum of rostroventral midline patterning defects [25]. The mutations are found in genes encoding components of the SHH pathway or in genes that, directly or indirectly, regulate *SHH* expression or pathway activity in the early midline or forebrain [4], [5], [16], [30], [32], [33].

Mouse models of HPE provide support for this model. We have shown that mice lacking the SHH co-receptor CDON display HPE with strain-dependent penetrance and severity, consistent with an important role for silent modifier genes in grading the spectrum of phenotypes [27], [28]. CDON is partially redundant with other co-receptors [27], [34], [35], and 129S6.*Cdon^−/−^* animals have only a substhreshold defect in SHH signaling that renders them sensitive to induction of HPE by additional insults, genetic or environmental, that are themselves insufficient to produce HPE [26]–[29], [34], [35].

Epidemiological evidence suggests that fetal alcohol exposure raises the likelihood of HPE [9]. We recently showed that in utero exposure to ethanol produces a nearly complete spectrum of HPE phenotypes in 129S6.*Cdon^−/−^* embryos, but is not teratogenic to 129S6.*Cdon^+/+^* embryos [29]. We hypothesized that a brief exposure to ethanol during gastrulation in a genetically sensitized embryo sets up a situation whereby the variable severity of HPE arises as individual embryos fail to reach a threshold level of SHH signaling at a specific developmental stage, with the stage of the deficit occurring stochastically and determining the severity of the outcome. This is consistent with observations in model systems demonstrating that SHH pathway activity is required during multiple stages of rostroventral midline patterning, including forebrain partitioning, bilateral division of the eye field and non-neural facial structures, and development of the primary and secondary palates [19], [36], [37].

This hypothesis argues that the level of SHH pathway signaling activity is an important determinant of phenotype. To provide experimental evidence for this possibility we tested the effect of removing one copy of the negative regulator *Ptch1* from *Cdon^−/−^* embryos and compared their response to ethanol-induced HPE with that of *Cdon^−/−^;Ptch1^+/+^* embryos. As predicted, reduced gene dosage of *Ptch1* substantially reduced the penetrance of HPE in this system, from ∼65% in *Cdon^−/−^;Ptch1^+/+^* embryos to ∼15% in *Cdon^−/−^;Ptch1^+/−^* embryos, a reduction of >75%. The major effect of *Ptch1* heterozygosity in this system was on penetrance, as those *Cdon^−/−^;Ptch1^+/−^* embryos that displayed HPE did not show major differences in phenotypic spectrum or selectivity from *Cdon^−/−^;Ptch1^+/+^* embryos with ethanol-induced HPE.

At what developmental stage(s) might the rescue of HPE by *Ptch1* haploinsufficiency occur? The PCM is required for induction of the ventral forebrain and subsequent partitioning of the forebrain and eye field, as well as patterning of the midfacial midline [1], [11]–[13]. The PCM serves as a source of SHH early during this multistep process, but SHH is also required for normal development of the PCM itself [1], [13], [14]. *Cdon* is expressed in the PCM but not in the ventral forebrain, even though the latter structure is affected in *Cdon^−/−^* mice; this observation and additional results argue that loss of *Cdon* function in the PCM is the most likely cause of HPE in such mice [26]-[28], [38]. CDON and PTCH1 bind to one another as components of the SHH receptor complex [7], [35] and, like *Cdon*, *Ptch1* is expressed in the PCM [14]. Therefore, it seems likely that rescue of HPE phenotypes in ethanol-treated *Cdon^−/−^;Ptch1^+/−^* embryos may occur as a consequence of enhanced SHH signaling function in the PCM itself. Alternatively, enhanced responsiveness to PCM-derived SHH may occur in the *Ptch1^+/−^* ventral forebrain, exclusive of co-expression of *Cdon* and *Ptch1*. Finally, *Cdon* and *Ptch1* are also co-expressed in the developing facial mesenchyme, and restoration of SHH signaling there may contribute to rescue of facial midline phenotypes. These possibilities are not mutually exclusive, and conditional mutagenesis will be required to address this issue. The mechanism whereby ethanol synergizes with *Cdon* mutation to inhibit SHH signaling is also not fully resolved but must involve early patterning events between E7.0 – E8.0, as ethanol is no longer detectable by E8.0 [29].

Studies on individuals with HPE and animal models conclusively demonstrate that HPE is associated with loss of SHH pathway function. The fact that haploinsufficiency for *Ptch1* rescues HPE phenotypes in ethanol-treated *Cdon^−/−^* embryos is consistent with PTCH1 functioning as a negative regulator of SHH signaling. However, heterozygous, germline missense mutations in *PTCH1* have been identified in HPE patients [39], [40]. PTCH1 functions in the absence of Hedgehog ligand to inhibit the activity of a second membrane protein, Smoothened (SMO). Binding of SHH to PTCH1 relieves this inhibition, and SMO signals to activate pathway target genes [41]. Because HPE is caused by loss, not gain, of SHH pathway function, it is highly unlikely that the *PTCH1* mutations found in HPE are complete loss-of-function mutations. In fact, heterozygous, germline loss-of-function mutations in *PTCH1* result in a different disorder, Basal Cell Nevus Syndrome (also called Gorlin syndrome) [42]. Rather, such PTCH1 variants would be predicted to maintain the ability to inhibit SMO activity but to be insensitive to ligand-induced pathway activation. A synthetically constructed deletion mutant with such properties is the widely used PTCH1^Δloop2^ variant [43].

Our findings are consistent with the notion that even in an etiologically complex animal model of HPE (one that models the complexities of human HPE), the level of SHH pathway activity is rate-limiting. These findings are highly relevant in the emerging era of human genomics and soon-to-be widespread personal access to genome sequences. Systematic analyses of common variants in SHH pathway components may reveal not only alleles that predispose offspring of heterozygous HPE mutation carriers to severe phenotypes but also alleles with protective effects. With a core database of variants – which will need to be functionally validated – it may be possible to predict the sum effect of deleterious and beneficial alleles on penetrance and expressivity of bona fide mutations and, conceivably, their interaction with non-genetic risk factors like fetal alcohol exposure.

## Materials and Methods

### Ethics Statement

All animal work was approved by the Icahn School of Medicine at Mount Sinai Institutional Animal Care and Use Committee (IACUC). Our animal facility is accredited by the Association for Assessment and Accreditation of Laboratory Animal Care International (AAALAC).

### Mice

STOCK.*Ptch1^tm1Mps/J^* mice [44] were purchased from the Jackson Laboratory and transferred onto the 129S6/SvEvTac (129S6) background with the Speed Congenics Program at Taconic, employing back-crossing and SNP arrays. Mice used for these experiments were estimated to be >98% 129S6. 129S6.*Ptch1^+/−^* mice were crossed with 129S6.*Cdon^+/−^* animals to generate 129S6.*Cdon^+/−^;Ptch1^+/−^* double mutant mice. These animals were then used to generate *Cdon^−/−^;Ptch1^+/−^*, *Cdon^+/−^;Ptch1^+/−^*, and *Cdon^+/−^;Ptch1^+/+^* mice, which were then variously crossed for ethanol treatment as previously described [29]. Briefly, two to three month-old mice were mated for one hour in the dark. The time of the plug was designated as embryonic day (E) 0.0. Pregnant female mice were injected intraperitoneally twice with 15 µl per g body weight of a solution of 30% ethanol in saline (3.48 g/kg), first at E7.0 and again 4 hr later. Saline injections were used as a control.

### Histology and Whole Mount In Situ Hybridization

Embryos were processed and analyzed as previously described [29]. Briefly, embryos were dissected out, fixed in 4% paraformaldehyde/PBS, dehydrated through a graded ethanol series, embedded in paraffin, and sectioned at 8 µm. H&E staining was performed as described [27]. Sections were dehydrated through graded ethanol and xylene and mounted with Permount (Fisher Scientific). For whole mount in situ hybridization, embryos were prepared as described previously [45], except that they were treated with 10 µg/ml proteinase K (QIAGEN) in phosphate-buffered saline, 0.1% Tween-20 (PBT) according to stage. Embryos were rinsed, postfixed and hybridized with digoxygenin-labeled probes in hybridization mix [50% formamide, 1.3x SSC, 5 mM EDTA, 50 µg/ml yeast RNA, 0.2% Tween 20, 0.5% 3-[(3-cholamidopropyl) dimethylammonio] propanesulfonate, and 100 µg/ml heparin] overnight at 65°C. They were then washed, blocked and incubated overnight with alkaline phosphatase-conjugated anti-digoxigenin antibody (1∶2000; Roche) in blocking buffer (2% blocking reagent [Roche]), 20% heat-inactivated lamb serum in 100 mM maleic acid, pH 7.5, 150 mM NaCl, and 0.1% Tween 20 [MABT]). After washing in TBST (Tris-buffered saline with 0.1% Tween-20) and NTMT (100 mm NaCl, 100 mm Tris-HCl, pH 9.5, 50 mm MgCl2, and 0.1% Tween -20), signals were developed with BM Purple AP Substrate (Roche). Embryos were photographed with a Jenoptik ProgRes C3 camera attached to a Nikon SMZ 1500 stereomicroscope. Captured images were assembled by Helicon Focus software (Helicon Soft).

## Supporting Information

Figure S1 **In utero exposure to saline does not induce external or forebrain features of HPE.** (A–D) Frontal views of E14.0 embryos. (E–H) H&E-stained coronal sections of E14.0 embryos. This figure serves as a control for Figure 1.
(TIF)

Figure S2 **In utero exposure to saline does not induce defects in palatogenesis or development of the midfacial midline** (A–L) H&E-stained coronal sections of E14.0 embryos. This figure serves as a control for Figure 2.
(TIF)

Figure S3 **In utero exposure to saline does not alter expression of** ***Shh*** **or** ***Nkx2.1*** **.** Whole mount in situ hybridization analyses of *Shh* (A–D) and *Nkx2.1* (E–H) expression in E10.25 (31–35 somites) saline-treated embryos of the indicated genotype. This figure serves as a control for Figure 3.
(TIF)

## References

[1] Muenke M, Beachy PA (2001) Holoprosencephaly. In: Scriver CR, Beaudet AL, Sly WS, Valle D, editors. The Metabolic & Molecular Bases of Inherited Disease. Eighth Edition ed. New York: McGraw-Hill. pp. 6203–6230.

[2] Orioli IM, Castilla EE (2010) Epidemiology of holoprosencephaly: Prevalence and risk factors. Am J Med Genet C Semin Med Genet 154C: 13–21. 20104599 10.1002/ajmg.c.30233

[3] Shiota K, Yamada S (2010) Early pathogenesis of holoprosencephaly. Am J Med Genet C Semin Med Genet 154C: 22–28. 20104600 10.1002/ajmg.c.30248

[4] Krauss RS (2007) Holoprosencephaly: new models, new insights. Expert Rev Mol Med 9: 1–17. 10.1017/S1462399407000440 17888203

[5] Roessler E, Muenke M (2010) The molecular genetics of holoprosencephaly. Am J Med Genet C Semin Med Genet 154C: 52–61. 20104595 10.1002/ajmg.c.30236 PMC2815021

[6] Solomon BD, Mercier S, Vélez JI, Pineda-Alvarez DE, Wyllie A, et al (2010) Analysis of genotype-phenotype correlations in human holoprosencephaly. Am J Med Genet C Semin Med Genet 154C: 133–141. 20104608 10.1002/ajmg.c.30240 PMC2815217

[7] Bae GU, Domené S, Roessler E, Schachter K, Kang JS, et al (2011) Mutations in CDON, Encoding a Hedgehog Receptor, Result in Holoprosencephaly and Defective Interactions with Other Hedgehog Receptors. Am J Hum Genet 89: 231–240. 21802063 10.1016/j.ajhg.2011.07.001 PMC3155179

[8] Ribeiro LA, Quiezi RG, Nascimento A, Bertolacini CP, Richieri-Costa A (2010) Holoprosencephaly and holoprosencephaly-like phenotype and GAS1 DNA sequence changes: Report of four Brazilian patients. Am J Med Genet A 152A: 1688–1694. 20583177 10.1002/ajmg.a.33466

[9] Johnson CY, Rasmussen SA (2010) Non-genetic risk factors for holoprosencephaly. Am J Med Genet C Semin Med Genet 154C: 73–85. 20104598 10.1002/ajmg.c.30242

[10] Miller EA, Rasmussen SA, Siega-Riz AM, Frías JL, Honein MA, et al (2010) Risk factors for non-syndromic holoprosencephaly in the National Birth Defects Prevention Study. Am J Med Genet C Semin Med Genet 154C: 62–72. 20104597 10.1002/ajmg.c.30244

[11] Kiecker C, Niehrs C (2001) The role of prechordal mesendoderm in neural patterning. Curr Opin Neurobiol 11: 27–33. 11179869 10.1016/s0959-4388(00)00170-7

[12] Muenke M, Beachy PA (2000) Genetics of ventral forebrain development and holoprosencephaly. Curr Opin Genet Dev 10: 262–269. 10826992 10.1016/s0959-437x(00)00084-8

[13] Rubenstein JL, Beachy PA (1998) Patterning of the embryonic forebrain. Curr Opin Neurobiol 8: 18–26. 9568388 10.1016/s0959-4388(98)80004-4

[14] Aoto K, Shikata Y, Imai H, Matsumaru D, Tokunaga T, et al (2009) Mouse Shh is required for prechordal plate maintenance during brain and craniofacial morphogenesis. Dev Biol 327: 106–120. 19103193 10.1016/j.ydbio.2008.11.022

[15] Cordero D, Marcucio R, Hu D, Gaffield W, Tapadia M, et al (2004) Temporal perturbations in sonic hedgehog signaling elicit the spectrum of holoprosencephaly phenotypes. J Clin Invest 114: 485–494. 15314685 10.1172/JCI19596 PMC506789

[16] Geng X, Speirs C, Lagutin O, Inbal A, Liu W, et al (2008) Haploinsufficiency of Six3 fails to activate Sonic hedgehog expression in the ventral forebrain and causes holoprosencephaly. Dev Cell 15: 236–247. 18694563 10.1016/j.devcel.2008.07.003 PMC2597207

[17] Marcucio RS, Cordero DR, Hu D, Helms JA (2005) Molecular interactions coordinating the development of the forebrain and face. Dev Biol 284: 48–61. 15979605 10.1016/j.ydbio.2005.04.030

[18] McMahon AP, Ingham PW, Tabin CJ (2003) Developmental roles and clinical significance of hedgehog signaling. Curr Top Dev Biol 53: 1–114. 12509125 10.1016/s0070-2153(03)53002-2

[19] Helms JA, Cordero D, Tapadia MD (2005) New insights into craniofacial morphogenesis. Development 132: 851–861. 15705856 10.1242/dev.01705

[20] Hu D, Helms JA (1999) The role of Sonic hedgehog in normal and abnormal craniofacial morphogenesis. Development 126: 4873–4884. 10518503 10.1242/dev.126.21.4873

[21] Hu D, Marcucio RS (2009) A SHH-responsive signaling center in the forebrain regulates craniofacial morphogenesis via the facial ectoderm. Development 136: 107–116. 19036802 10.1242/dev.026583 PMC2685963

[22] Cohen MM Jr (2006) Holoprosencephaly: clinical, anatomic, and molecular dimensions. Birth Defects Res Part A Clin Mol Teratol 76: 658–673. 17001700 10.1002/bdra.20295

[23] Ming JE, Muenke M (2002) Multiple hits during early embryonic development: digenic diseases and holoprosencephaly. Am J Hum Genet 71: 1017–1032. 12395298 10.1086/344412 PMC385082

[24] Mercier S, Dubourg C, Garcelon N, Campillo-Gimenez B, Gicquel I, et al (2011) New findings for phenotype-genotype correlations in a large European series of holoprosencephaly cases. J Med Genet 48: 752–760. 21940735 10.1136/jmedgenet-2011-100339 PMC3386902

[25] Roessler E, Vélez JI, Zhou N, Muenke M (2012) Utilizing prospective sequence analysis of SHH, ZIC2, SIX3 and TGIF in holoprosencephaly probands to describe the parameters limiting the observed frequency of mutant gene×gene interactions. Mol Genet Metab 105: 658–664. 22310223 10.1016/j.ymgme.2012.01.005 PMC3309119

[26] Tenzen T, Allen BL, Cole F, Kang J-S, Krauss RS, et al (2006) The cell surface membrane proteins Cdo and Boc are components and targets of the hedgehog signaling pathway and feedback network in mice. Dev Cell 10: 647–656. 16647304 10.1016/j.devcel.2006.04.004

[27] Zhang W, Hong M, Bae G-U, Kang J-S, Krauss RS (2011) *Boc* modifies the holoprosencephaly spectrum of *Cdo* mutant mice. Dis Model Mech 4: 368–380. 21183473 10.1242/dmm.005744 PMC3097458

[28] Zhang W, Kang J-S, Cole F, Yi MJ, Krauss RS (2006) Cdo functions at multiple points in the Sonic Hedgehog pathway, and Cdo-deficient mice accurately model human holoprosencephaly. Dev Cell 10: 657–665. 16647303 10.1016/j.devcel.2006.04.005

[29] Hong M, Krauss RS (2012) *Cdo*n mutation and fetal ethanol exposure synergize to produce midline signaling defects and holoprosencephaly spectrum disorders in mice PLoS Genet. 8: e1002999. 10.1371/journal.pgen.1002999 PMC3469434 23071453

[30] Ingham PW, Nakano Y, Seger C (2011) Mechanisms and functions of Hedgehog signalling across the metazoa. Nat Rev Genet 12: 393–406. 21502959 10.1038/nrg2984

[31] Cole F, Krauss RS (2003) Microform holoprosencephaly in mice that lack the Ig superfamily member Cdon. Curr Biol 13: 411–415. 12620190 10.1016/s0960-9822(03)00088-5

[32] Taniguchi K, Anderson AE, Sutherland AE, Wotton D (2012) Loss of Tgif function causes holoprosencephaly by disrupting the SHH signaling pathway. PLoS Genet 8: e1002524. 22383895 10.1371/journal.pgen.1002524 PMC3285584

[33] Warr N, Powles-Glover N, Chappell A, Robson J, Norris D, et al (2008) Zic2-associated holoprosencephaly is caused by a transient defect in the organizer region during gastrulation. Hum Mol Genet 17: 2986–2996. 18617531 10.1093/hmg/ddn197

[34] Allen BL, Song JY, Izzi L, Althaus IW, Kang J-S, et al (2011) Overlapping roles and collective requirement for the co-receptors Gas1, Cdo and Boc in Shh pathway function. Dev Cell 20: 775–787. 21664576 10.1016/j.devcel.2011.04.018 PMC3121104

[35] Izzi L, Lévesque M, Morin S, Laniel D, fWilkes BC, et al (2011) Boc and Gas1 each form distinct Shh receptor complexes with Ptch1 and are required for Shh-mediated cell proliferation. Dev Cell 20: 788–801. 21664577 10.1016/j.devcel.2011.04.017 PMC3432913

[36] Marcucio RS, Young NM, Hu D, Hallgrimsson B (2011) Mechanisms that underlie co-variation of the brain and face. Genesis 49: 177–189. 21381182 10.1002/dvg.20710 PMC3086711

[37] Schachter KA, Krauss RS (2008) Murine models of holoprosencephaly. Curr Top Dev Biol 84: 139–170. 19186244 10.1016/S0070-2153(08)00603-0

[38] Mulieri PJ, Okada A, Sassoon DA, McConnell SK, Krauss RS (2000) Developmental expression pattern of the *cdo* gene. Dev Dyn 219: 40–49. 10974670 10.1002/1097-0177(2000)9999:9999<::AID-DVDY1032>3.0.CO;2-M

[39] Ming JE, Kaupas ME, Roessler E, Brunner HG, Golabi M, et al (2002) Mutations in PATCHED-1, the receptor for SONIC HEDGEHOG, are associated with holoprosencephaly. Hum Genet 110: 297–301. 11941477 10.1007/s00439-002-0695-5

[40] Ribeiro LA, Murray JC, Richieri-Costa A (2006) PTCH mutations in four Brazilian patients with holoprosencephaly and in one with holoprosencephaly-like features and normal MRI. Am J Med Genet A 140: 2584–2586. 17001668 10.1002/ajmg.a.31369

[41] Jiang J, Hui CC (2008) Hedgehog signaling in development and cancer. Dev Cell 15: 801–812. 19081070 10.1016/j.devcel.2008.11.010 PMC6443374

[42] Lindström E, Shimokawa T, Toftgård R, Zaphiropoulos PG (2006) PTCH mutations: distribution and analyses. Human Mutation 27: 215–219. 16419085 10.1002/humu.20296

[43] Briscoe J, Chen Y, Jessell TM, Struhl G (2001) A hedgehog-insensitive form of patched provides evidence for direct long-range morphogen activity of sonic hedgehog in the neural tube. Mol Cell 7: 1279–1291. 11430830 10.1016/s1097-2765(01)00271-4

[44] Goodrich LV, Milenković L, Higgins KM, Scott MP (1997) Altered neural cell fates and medulloblastoma in mouse patched mutants. Science 277: 1109–1113. 9262482 10.1126/science.277.5329.1109

[45] Mulieri PM, Kang J-S, Sassoon DA, Krauss RS (2002) Expression of the *boc* gene during murine embryogenesis. Dev Dyn 223: 379–388. 11891987 10.1002/dvdy.10063

