# Supplementary figures and images for: Rescue of Holoprosencephaly in Fetal Alcohol-Exposed Cdon Mutant Mice by Reduced Gene Dosage of Ptch1
Source: PLoS One. 2013 Nov 11;8(11):e79269. doi: 10.1371/journal.pone.0079269 (PMC3823703; doi:10.1371/journal.pone.0079269)

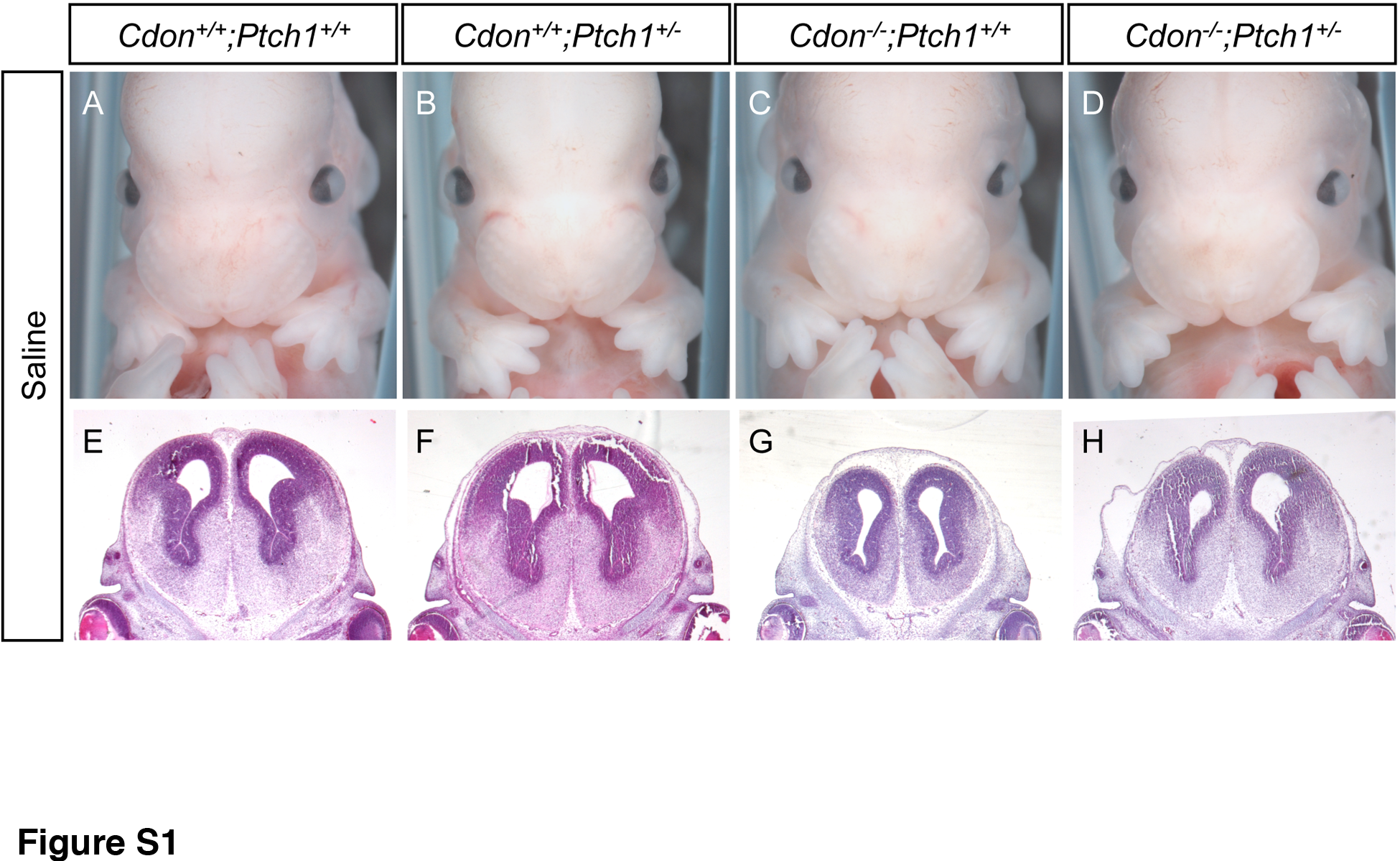

Supplement: Figure S1 — In utero exposure to saline does not induce external or forebrain features of HPE. (A–D) Frontal views of E14.0 embryos. (E–H) H&E-stained coronal sections of E14.0 embryos. This figure serves as a control for Figure 1. (TIF) [file pone.0079269.s001.tif]

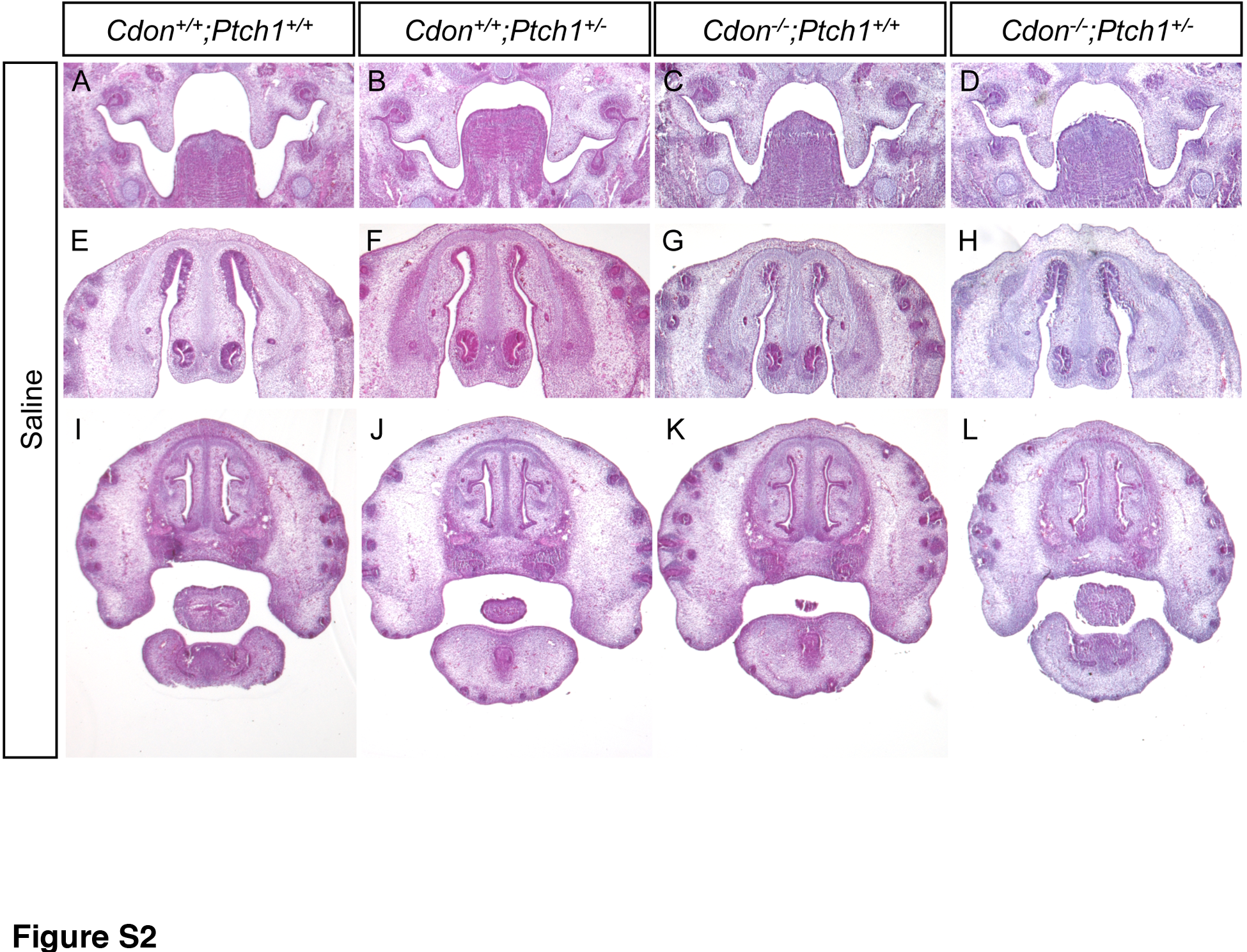

Supplement: Figure S2 — In utero exposure to saline does not induce defects in palatogenesis or development of the midfacial midline (A–L) H&E-stained coronal sections of E14.0 embryos. This figure serves as a control for Figure 2. (TIF) [file pone.0079269.s002.tif]

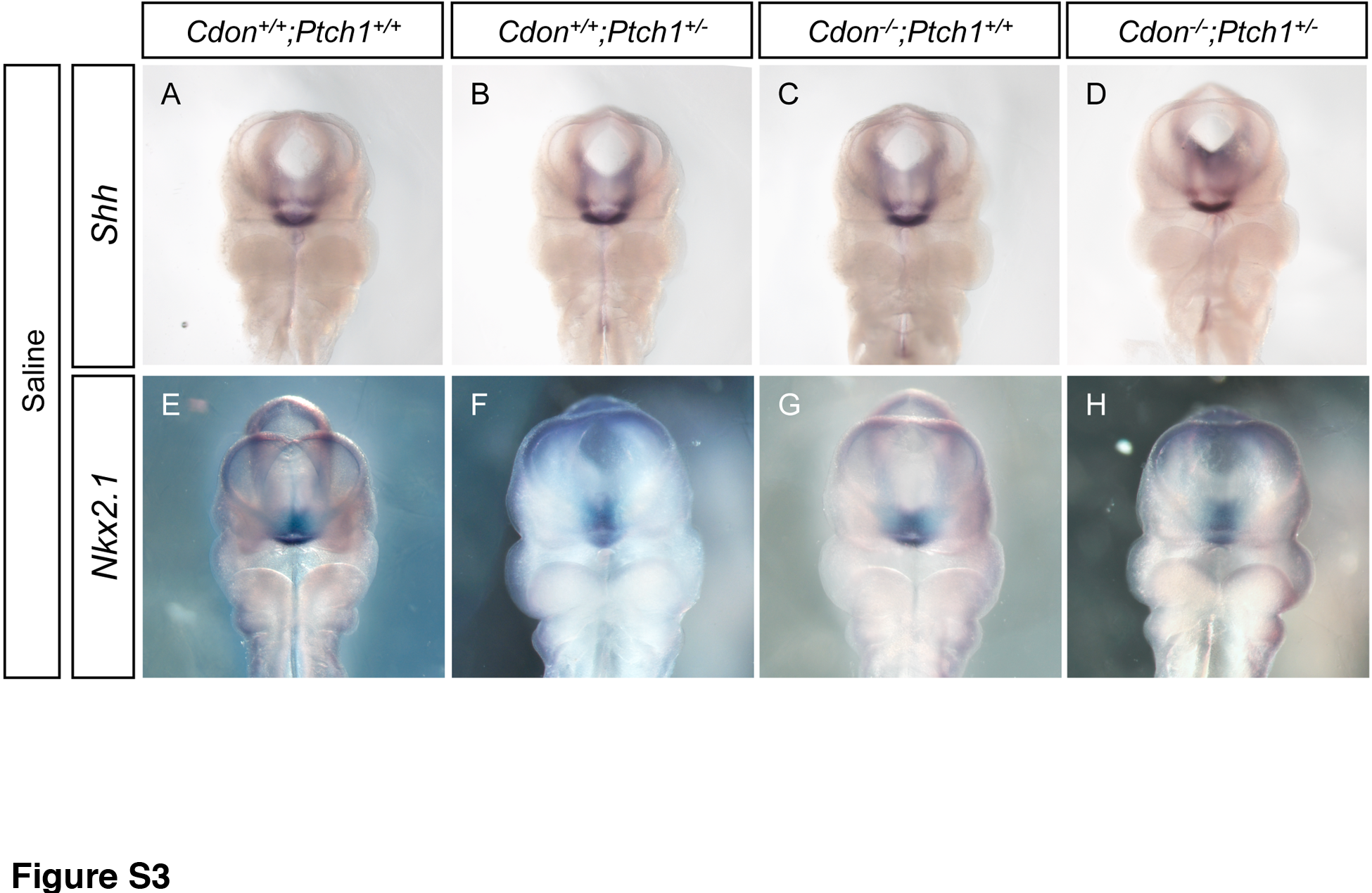

Supplement: Figure S3 — In utero exposure to saline does not alter expression of Shh or Nkx2.1 . Whole mount in situ hybridization analyses of Shh (A–D) and Nkx2.1 (E–H) expression in E10.25 (31–35 somites) saline-treated embryos of the indicated genotype. This figure serves as a control for Figure 3. (TIF) [file pone.0079269.s003.tif]
